# Influence of teach-back strategy on hemodialysis related knowledge level, self-efficacy and self-management in patients receiving maintenance hemodialysis

**DOI:** 10.1038/s41598-024-54044-6

**Published:** 2024-02-18

**Authors:** Fuhai Xia, Guoqing Wang

**Affiliations:** Hemodialysis Center, Renmin Hospital of Wuhan University, Wuhan City, 430060 Hubei Province China

**Keywords:** Teach-back, Hemodialysis, Self-efficacy, Self-management, Health education, Haemodialysis, Patient education

## Abstract

To investigate the effect of teach-back strategy on hemodialysis related knowledge level, self-efficacy and self-management among hemodialysis patients. The research is a quasi-randomized control trial. A total of 92 patients receiving maintenance hemodialysis were randomly divided into observation group (n = 46) and control group (n = 46) by random number table method. The control group received conventional health education, and the observation group received teach-back. The intervention lasted six months. The hemodialysis related knowledge level, self-efficacy and self-management of the two groups were evaluated before and after the intervention. There were no significant difference on hemodialysis related knowledge level, self-efficacy and self-management scores between the two groups before intervention (*P* > 0.05). After intervention, the scores of hemodialysis knowledge in the observation group was higher than that in the control group and before intervention. The total scores of self-efficacy and items “3,4,5” were higher than those of the control group. The total scores of self-efficacy and item “1~6” in the observation group were higher than before intervention. The total scores of self-management and the three subscales of “problem solving”, “partnership” and “emotional processing” were higher than those of the control group and before intervention. All of the above differences were statistically significant (*P* < 0.05). Teach-back is helpful to improve the hemodialysis related knowledge level, self-efficacy and self-management level of patients receiving maintenance hemodialysis, and it is worth to be popularized clinically.

## Introduction

Chronic kidney disease (CKD) has become a serious public health problem worldwide, and about 2% of patients progress to end-stage renal disease (ESRD) every year^1,2^. As the main treatment of ESRD, maintenance hemodialysis (MHD) is widely used in clinic. Patients receiving MHD have the characteristics of long disease cycle, frequent and regular dialysis, and more dialysis-related complications. According to the United States Renal Data System's 2021 annual data release, the near-uniform linear increase in the prevalent count of end-stage renal disease (ESRD) since 2009^3^. It has been reported that the number of patients receiving hemodialysis in China has reached nearly 700,000^2^. This group has gradually become the focus and hot spot of social attention.

According to social cognitive theory (SCT), self-efficacy, defined as one's confidence in performing a behavior, is an important determinant of initiating and maintaining certain behaviors^4^. It has been reported that the self-efficacy level of patients receiving MHD is closely related to their positive beliefs about disease, health outcomes, treatment compliance and satisfaction^5^. Self-management is when individuals develop action plans based on perceived problems to establish achievable health care goals^6^. The improvement of self-management in patients receiving MHD can improve their coping ability, symptom management, quality of life and reduce medical costs^7^. Therefore, the implementation of effective intervention program is of great significance for patients receiving MHD to manage their own health problems.

Teach-back, as a strategy superior to conventional health education, has been widely used and has achieved certain effects^8,9^. Teach-back means that healthcare provider guide patients to repeat (in their own words) the information they have received after health education. Based on the results of patient feedback, the provider then corrects and reinterprets the information that the patient does not understand or understands ambiguates until the patient can understand and master the relevant knowledge^10^. The benefits of teach-back have not yet previously been studied in patients receiving MHD. In this study, teach-back was used to intervene patients receiving MHD and its effect was observed.

## Methods

### Sample

The research is a quasi-randomized control trial. From November 2018 to October 2019, 92 convenience samples were recruited from the Hemodialysis Centers of a tertiary hospitals in Wuhan, Hubei Province, China. Participants could have been considered if they matched the following criteria: (1) at least 3 months of MHD treatment, (2) at least 18 years of age, (3) no cognitive or communication problems, (4) informed consent and willingness to engage in the study. Participants may be excluded if they transferred to transplantation, peritoneal dialysis, and lost to follow-up.

Sample size was estimated by PASS version 15.0. Based on the result of similar study^11^, the effect rates of the control group and the observation group were calculated with a 0.75 effect size. The α was 0.05 and the power was 0.90. The study should have included 76 patients. Given a 15% sample loss, the sample size should be 88, which means 92 samples match the criteria. We recruited 100 participants, but 8 participants were excluded due to language barrier, so 92 participants finally met the inclusion criteria. According to the random number table, 92 convenience samples were divided into observation group and control group, 46 cases in each group. The control group received conventional health education, and the observation group received teach-back.

### Setting

Control group: Conventional health education. Researchers used WeChat official account and Tencent QQ platform to conduct individualized health education for participants. The contents include the basic knowledge of hemodialysis, drug guidance, fluid intake, diet guidance of low phosphorus and low potassium, the significance of regular dialysis and regular examination, vascular access nursing, etc. The form of health education is mainly taught by researchers. Patients were asked questions every two weeks for 1 to 1.5 h 12 times over a period of 6 months. The question-and-answer session was conducted during hemodialysis treatment.

Observation group: Teach-back. The research team consists of 1 doctor, 1 head nurse, 2 national nurses specializing in blood purification and 2 graduate students. The research team members uniformly received the training of teach-back related theories and implementation skills, and were assessed after the training. Doing so would ensure homogeneity in the implementation of the intervention. An accessible education manual suitable for patients receiving MHD was developed and revised after repeated discussion by our research team. The researchers divided 46 participants into four groups of 10 to 12 people each. The intervention was administered every two weeks for 1 to 1.5 h and 12 times over 6 months. The intervention site was in the hemodialysis reception room.

### Intervention procedure

Teach-back consists of the following five steps: (1) Convey information. Researchers should be careful to use non-medical terms, speak at a moderate pace, and step by step when explaining or demonstrating hemodialysis related knowledge to patients in accordance with the content of the health education program. Illustrations, multimedia and other auxiliary tools can be used to explain. The researchers selected two to three topics of 20–25 min each and informed the patients about the significance of the topics. (2) Repeat information. After the researchers' health education, patients were asked to repeat the information in their own words. In the field demonstration part, researchers should pay attention to create a relaxed atmosphere and ask questions in a moderate tone. The time is 15 to 20 min each time. (3) Effect evaluation. After the patients repeated or demonstrated the information, the researchers evaluated the effect of the repeated information to understand the patients' understanding and mastery of the information knowledge. The time is 5–10 min each time. (4) Clarification. Patients did not understand or understood the content of ambiguity, the researchers used different words or tools to explain and clarify again. If the patient could not understand correctly after 2 or 3 times of clarification by the researcher, the patient should change other health education methods to explain until the patient could understand correctly. The time is 15–20 min each time. (5) Open-ended questions. Patients were asked open-ended questions at the end of the session, such as “Is there anything you don't understand?”. If the patient can correctly and comprehensively understand the content of health education, then the health education is finished, otherwise, repeat steps 1 to 5 until the patient has fully grasped. The time is 5 to 10 min each time.

### Measures

Hemodialysis related knowledge scale, self-efficacy for managing chronic disease 6-item scale and self-management scale for hemodialysis patients were used to evaluate the hemodialysis related knowledge level, self-efficacy and self-management of patients receiving MHD before and after intervention (Baseline, after 6 months of intervention).

Hemodialysis related knowledge scale^12^. The scale is used to evaluate the knowledge level of hemodialysis in patients receiving MHD. A two-point Likert scale (0 = “wrong answer or don't know” to 1 = “correct answer”) is used and the total score of 25 items varies from 0 to 25, with higher scores suggesting a higher level of knowledge. The instrument has been proven to be valid and reliable, and has been used among Chinese hemodialysis patients^13^. In this study, the Cronbach’s alpha coefficient was 0.701.

Self-efficacy for managing chronic disease 6-item scale^14^. The scale includes 2 subscales: symptom management (fatigue, shortness of breath, pain, role function, depression, and health distress) and common disease management (take medicine as prescribed and adopt health care behaviors). A visual analogue scale (VAS) (1 = “have no confidence” to 10 = “have every confidence”) is used and the total score of 6 items varies from 6 to 60, with higher scores suggesting a higher level of self-efficacy. In this study, the Cronbach’s alpha coefficient was 0.910.

Self-management scale for hemodialysis patients. The scale includes 4 subscales: problem solving, perform self-care activities, partnership and emotional processing. A four-point Likert scale (1 = “never” to 4 = “always”) is used and the total score of 20 items varies from 20 to 80, with higher scores suggesting a higher level of self- management. The instrument was developed by Chinese scholars and has proved to be valid and reliable^15^. In this study, the Cronbach’s alpha coefficient was 0.811, and the Cronbach’s alpha of 4 subscales ranged from 0.736 to 0.829.

### Data analysis

SPSS version 26.0 (IBM Corporation, Armonk, New York, USA) were used to analyze the data. PASS version 15.0 was used to estimate the sample size. Frequency and percentage were used to describe categorical variables. Mean and standard deviation (SD) were used to describe continuous variables. Chi-square test (χ^2^) was used for comparison between groups. 2-Independent-samples *t* test was used to compare the two groups before and after intervention. The paired *t* test was used to compare the self before and after. Kolmogorov–Smirnov test was used for normality test. All statistical tests were conducted by two-sided tests, and *p* values of < 0.05 indicated statistical significance.

### Ethical consideration

All operations involving human volunteers were approved by the hospital ethics committee, and the study followed the principles of Declaration of Helsinki. Informed consent was obtained from patients and their families during the study. After the study, the control group was also given teach-back.

### Ethics approval and consent to participate

This study was approved by the ethics committee of Renmin Hospital of Wuhan University (number WDRY2022-K192). Every method was used in accordance with the relevant rules and regulations of the Declaration of Helsinki. All participants gave their voluntary written informed consent prior to study participation.

## Result

### Sample characteristics

**Figure 1:**
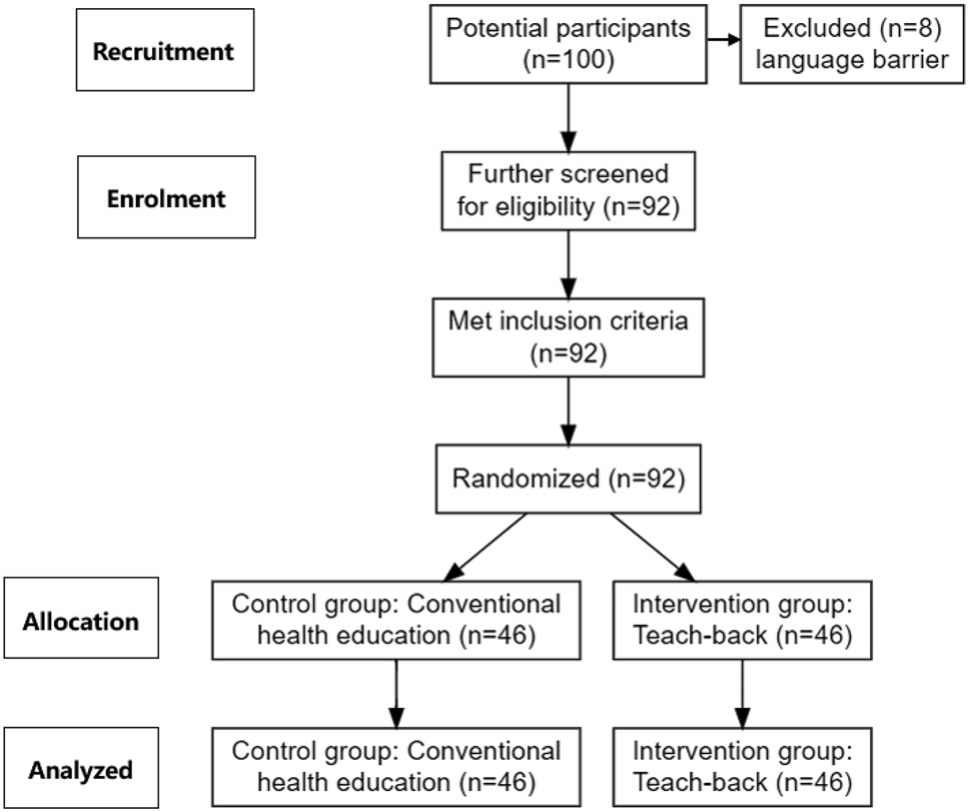
Participant flowchart.

We recruited 100 participants, but 8 participants were excluded due to language barrier, so 92 participants finally met the inclusion criteria. Figure 1 shows the participant recruitment process. There was no significant difference in the sample characteristics between the two groups of patients receiving MHD (*P* > 0.05). More information about sample characteristics is reported in Table 1.

**Table 1:** Sample characteristics.

Variables	Observation group	Control group	*t/χ*^*2*^ value	*P* value
Age	53.5 ± 14.1*	57.5 ± 12.6*	1.420	0.159
Gender			1.592	0.207
Male	29	23		
Female	17	23		
Education			3.458	0.177
Primary	12	18		
Secondary	19	11		
Higher	15	17		
Average income per month (USD)			0.865	0.649
< 444	21	17		
444–740	16	20		
> 740	9	9		
Marital status			0.080	0.778
Married	38	39		
Single or divorced	8	7		
Primary disease			1.880	0.598
Chronic glomerulonephritis	10	9		
Hypertensive kidney lesion	18	19		
Diabetic nephropathy	6	10		
Other disease	12	8		

*Represents Mean ± SD.

### Comparison of hemodialysis related knowledge scores between the two groups before and after intervention

After 6 months of intervention, the scores of hemodialysis-related knowledges in the observation group were not only higher than those in the control group, but also higher than those in the observation group at baseline. The differences were statistically significant (*P* < 0.05). Table 2 shows the comparison of hemodialysis related knowledge scores between the two groups before and after intervention.

**Table 2:** Comparison of hemodialysis related knowledge scores between the two groups before and after intervention.

Variables	Baseline				After 6 months of intervention			
	Observation group	Control group	*t* value	*P* value	Observation group	Control group	*t* value	*P* value
Hemodialysis related knowledge	14.43 ± 3.79	14.84 ± 3.39	− 0.550	0.584	20.45 ± 2.50*	15.93 ± 2.36	8.901	0.000

### Comparison of self-efficacy scores between the two groups before and after intervention

After 6 months of intervention, the total score of self-efficacy and the scores of item “3,4,5” in the observation group were higher than those in the control group. The total score of self-efficacy and the scores of items "1–6" in the observation group were higher than those before the intervention. These differences were statistically significant (*P* < 0.05). Table 3 compares the self-efficacy scores between the two groups before and after intervention.

**Table 3:** Comparison of self-efficacy scores between the two groups before and after intervention.

Variables	Baseline				After 6 months of intervention			
	Observation group	Control group	*t* value	*P* value	Observation group	Control group	*t* value	*P* value
1. Confidence in managing fatigue	6.71 ± 2.25	6.50 ± 2.28	0.459	0.648	7.26 ± 1.38*	6.60 ± 2.19	1.702	0.092
2. Confidence in managing physical discomfort or pain	6.23 ± 2.12	6.34 ± 2.12	− 0.246	0.806	6.73 ± 1.52*	6.45 ± 2.07	0.744	0.459
3. Confidence in managing depression	6.67 ± 2.30	6.60 ± 2.32	0.135	0.893	7.41 ± 1.12*	6.39 ± 2.05	2.951	0.004
4. Confidence in managing health problems	6.23 ± 2.22	6.30 ± 2.29	− 0.138	0.890	7.34 ± 1.30*	6.32 ± 1.85	3.062	0.003
5. Confidence in adopting self-care behaviors	6.41 ± 2.50	6.06 ± 2.36	0.685	0.495	7.06 ± 1.71*	6.19 ± 2.31	2.046	0.044
6. Confidence in reducing the impact of illness on life	6.43 ± 2.61	6.36 ± 2.22	0.129	0.898	6.78 ± 2.24*	6.45 ± 2.09	0.721	0.473
Total score of self-efficacy	38.71 ± 12.09	38.19 ± 11.73	0.210	0.834	42.60 ± 6.97*	38.43 ± 10.77	2.205	0.030

### Comparison of self-management scores between the two groups before and after intervention

After 6 months of intervention, the total score of self-management and three subscales (problem solving, partnership and emotional processing) in the observation group were higher than those in the control group and before intervention. These differences were statistically significant (*P* < 0.05). Table 4 compares the self-management scores between the two groups before and after intervention.

**Table 4:** Comparison of self-management scores between the two groups before and after intervention.

Variables	Baseline				After 6 months of intervention			
	Observation group	Control group	*t* value	*P* value	Observation group	Control group	*t* value	*P* value
Problem solving	14.80 ± 3.44	14.50 ± 3.06	0.448	0.655	16.76 ± 2.23*	14.52 ± 2.62	4.409	0.000
Perform self-care activities	17.91 ± 4.52	18.45 ± 3.91	− 0.616	0.539	18.39 ± 3.85	18.47 ± 3.82	− 0.109	0.914
Partnership	12.21 ± 2.64	12.06 ± 2.45	0.286	0.776	14.39 ± 1.85*	12.23 ± 2.30	4.937	0.000
Emotional processing	9.93 ± 2.28	10.39 ± 2.64	− 0.886	0.378	13.04 ± 1.89*	10.54 ± 2.54	5.342	0.000
Total score of self-management	54.86 ± 10.26	55.41 ± 9.91	− 0.258	0.797	62.58 ± 7.05*	55.78 ± 9.23	3.971	0.000

*Represents comparison with self before intervention, *P* < 0.05.

## Discussion

Teach-back strategy is in line with the concept of the “learning pyramid”^16^. In the process of health education, researchers mobilize the enthusiasm of patients to learn by drawing, video, group discussion, retelling and practical exercise, so that patients can master relevant knowledge through active participation. The results of this study show that teach-back has obvious advantages over conventional health education in improving the hemodialysis related knowledge level of patients receiving MHD. The reasons are as follows: First, it has been reported that 40% ~ 80% of the medical information delivered to patients by conventional health education will be forgotten by them, and 50% of the information saved in patients' memory is wrong^17^. Teach-back overcomes the disadvantages of simple form and one-way transmission, and pays more attention to two-way communication with patients^18^. Second, after receiving the knowledge, patients gave feedback, which improved their initiative to participate. Third, the researchers evaluated the patients after their feedback, and the knowledge that the patients did not master was taught again until they fully grasped it. Four, the intervention strategy of centralized learning makes patients discuss with each other, which enhances the efficiency of health education. It suggests that medical staff should translate the professional knowledge of hemodialysis into plain language when conducting health education for patients, so as to stimulate the initiative and interest of patients receiving MHD in learning.

The self-efficacy and self-management level of patients receiving MHD before intervention in this study were similar to the previous study result^19^, which suggests that there is still much room for improvement in self-efficacy and self-management level. After six months of teach-back intervention, the self-efficacy and self-management level of patients receiving MHD were significantly improved. It has been reported that the hemodialysis related knowledge level of patients receiving MHD was positively correlated with self-efficacy and self-management^20–22^. With the improvement of patients' knowledge level, their confidence and ability to manage their own symptoms are enhanced, so as to improve their self-efficacy and self-management level. The results of this study showed that after 6 months of intervention, the scores of items “1,2,6” in the self-efficacy scale in the observation group were not statistically significant compared with those in the control group. This may be related to the long disease cycle and many dialysis complications in patients receiving MHD, which are difficult to improve in the short term, such as fatigue, physical discomfort, and the impact on daily life. The results of this study showed that after 6 months of intervention, the score of “perform self-care activities” in the self-management scale in the observation group were not statistically significant compared with those in the control group. This may be related to the fact that the study intervention was limited to the education of knowledge and paid less attention to perform self-care activities. It suggests that medical staff should not only give theoretical knowledge education, but also strengthen the mastery of patients' self-care skills when carrying out health education for patients. At the same time, healthcare providers should guide patients on regular dialysis, proper exercise and nutrition to reduce their complications, and give appropriate psychological counseling to improve their confidence and ability, so as to further improve their self-efficacy and self-management level.

This study has several limitations. First, the data were collected from single center, which might not be representative of all patients receiving MHD. Second, the intervention time was short, and there was no long-term health education. Third, this study only conducted random grouping, not random sampling. In the future, it will be interesting to further explore the applicability and intervention effect of teach-back in health education for patients receiving MHD by increasing study samples, extending the intervention time, and improving the study design.

In conclusion, this study applied the teach-back strategy to the health education of patients receiving MHD, which is scientific, effective, low-cost, and could strongly improve their self-efficacy and self-management level. The improvement of self-efficacy and self-management level will help to improve the prognosis and quality of life of patients receiving MHD, and prolong the duration of dialysis.

## Data Availability

The datasets used and/or analysed during the current study available from the corresponding author on reasonable request.

## Abbreviations

CKD: Chronic kidney disease
ESRD: End-stage renal disease
MHD: Maintenance hemodialysis
SCT: Social cognitive theory
SD: Standard deviation
